# Role of Flavonoids in the Treatment of Iron Overload

**DOI:** 10.3389/fcell.2021.685364

**Published:** 2021-07-05

**Authors:** Xiaomin Wang, Ye Li, Li Han, Jie Li, Cun Liu, Changgang Sun

**Affiliations:** ^1^College of First Clinical Medicine, Shandong University of Traditional Chinese Medicine, Jinan, China; ^2^Shandong Academy of Chinese Medicine, Jinan, China; ^3^College of Traditional Chinese Medicine, Shandong University of Traditional Chinese Medicine, Jinan, China; ^4^Department of Oncology, Weifang Traditional Chinese Hospital, Weifang, China; ^5^Qingdao Academy of Chinese Medical Sciences, Shandong University of Traditional Chinese Medicine, Qingdao, China

**Keywords:** flavonoids, iron overload, iron metabolism, iron balance, plant iron chelator

## Abstract

Iron overload, a high risk factor for many diseases, is seen in almost all human chronic and common diseases. Iron chelating agents are often used for treatment but, at present, most of these have a narrow scope of application, obvious side effects, and other disadvantages. Recent studies have shown that flavonoids can affect iron status, reduce iron deposition, and inhibit the lipid peroxidation process caused by iron overload. Therefore, flavonoids with iron chelating and antioxidant activities may become potential complementary therapies. In this study, we not only reviewed the research progress of iron overload and the regulation mechanism of flavonoids, but also studied the structural basis and potential mechanism of their function. In addition, the advantages and disadvantages of flavonoids as plant iron chelating agents are discussed to provide a foundation for the prevention and treatment of iron homeostasis disorders using flavonoids.

## Introduction

Iron overload is a long-standing problem that has aroused great interest in the field of chronic diseases (Fernández-Real and Manco, 2014). Iron, one of the essential metal elements, maintains normal physiological activities in human body, which is mainly involved in important physiological processes such as oxygen transport, electron transport, DNA synthesis, and many enzymatic reactions. Iron is absorbed through the gastrointestinal tract, delivered into the bloodstream, distributed throughout the body, and there are no physiologically routes of excretion after absorption (Fleming and Ponka, 2012). When there is too much iron in the body, excess iron can be deposited in tissues and organs, produce lipid peroxidation, affects cell damage, which can lead to cancer, hematological diseases, and other chronic and commonly encountered diseases. In addition, iron overload is a characteristic of ferroptosis that is a form of regulated cell death, leading to accumulation of lethal levels of lipid hydroperoxides (Wu et al., 2021). The review of iron overload is helpful for us to better understand the occurrence and progress mechanism of ferroptosis.

Studies on iron overload mainly focus on liver fibrosis, liver cancer (Molina-Sánchez and Lujambio, 2019), atherosclerosis (Wunderer et al., 2020), and hematological diseases (Franke et al., 2020). Deferrioxamine, deferiprone, and deferasirox are usually used to treat iron overload as iron chelators, but they are prone to side effects. Flavonoids, with their special structure, maybe have good iron chelation, antioxidant properties and less toxic. Based on this, flavonoids have become a hot topic as plant iron chelators.

Recent researches on the treatment of iron overload by flavonoids mainly concentrated on the effect of animal or cell experiments of a single compound, and there was a lack of macroscopic summing up and sorting out from the same kind of compounds. The mechanisms involved in different studies are different or complicated, some of the experimental results are controversial and contradictory, and lack of systematic summary. In this paper, the research progress on the regulation mechanism of iron overload and flavonoids was reviewed, and the potential molecular cytological mechanism was generalized according to their structural basis. These findings provide a solid evidence base for flavonoids as plant iron chelating agents.

## Iron Overload: An Imbalance Iron Metabolism That Causes Chronic and Common Diseases

### Iron Metabolism of Iron Overload

**FIGURE 1:**
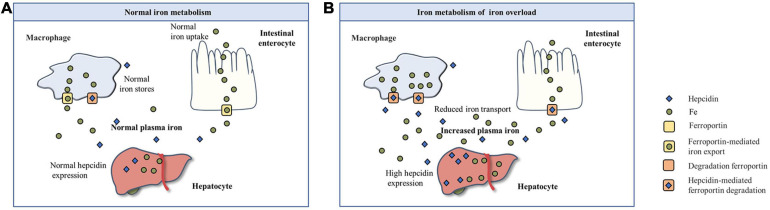
The basic process of iron metabolism. **(A)** Normal iron metabolism. **(B)** Iron metabolism of iron overload.

Iron metabolism is a process in which iron is absorbed, regulated in the organism, and eventually excreted (Figure 1A). Fe^3+^ in the intestinal cavity is reduced to Fe^2+^ by duodenal cytochrome B, and then transported by divalent metal ion transporter 1 to the absorption cells of the intestinal mucosa epithelium. Some of this combines with apoferritin to form ferritin, which is stored in cells, and the remaining Fe^2+^ is released from the basal end of absorbing cells and enters the blood circulation, mediated by ferroportin (FPN), and is oxidized to Fe^3+^ by hephaestin. Excess ferritin is lost when cells are shed in the gut (Ramakrishnan et al., 2018). At the same time, the regulation of iron involves the combined action of various proteins and pathways. Among these, hepcidin plays a central regulatory role in the maintenance of iron homeostasis (Lee and Beutler, 2009).

Iron overload or iron deficiency are the mainly iron dyshomeostasis (Muñoz et al., 2011). Iron deficiency is the most common public nutrition problem worldwide (Pasricha et al., 2020), but less attention is paid to iron overload. Iron overload is a pathological phenomenon in which the supply of iron in the body exceeds the need for iron, which leads to an increase in iron storage in some tissues and organs (Sousa et al., 2020). The human body has many absorption, transport, and storage mechanisms for iron, but there is no mechanism for excretion of excessive iron. Excessive iron can also have toxic effects on the body. An increasing amount of studies have shown that iron overload is related to the occurrence and development of many diseases. With respect to iron metabolism in the case of iron overload (Figure 1B), free iron exceeds the binding limit of transferrin (Tf), and iron absorption efficiency decreases relatively, but absolute values increase. At the same time, hepcidin levels increase, thereby accelerating the degradation of FPN, closing the iron transport outlet to the blood, and reducing the transport of iron from small intestinal epithelial cells and macrophages to the blood (Yan et al., 2018). Iron overload in the body can stimulate the synthesis of ferritin and capture excessive iron (Pickart et al., 2014).

In addition, ferroptosis is closely related to iron metabolism under iron overload. Ferroptosis, as a kind of programmed apoptosis, is characterized by excessive accumulation of lipid peroxides and reactive oxygen species (Zhai et al., 2021). Ferroptosis, often accompanied with iron overload, caused the tissue damage mainly driven by iron overload and lipid peroxidation. At the same time, ferroptosis causes ferritin degradation, affects iron metabolism and leads to iron overload (Chen et al., 2021). The occurrence of ferroptosis leads to normal tissue and organ damage and loss of function, which is directly involved in the occurrence, development and prognosis of some chronic and common diseases (Mao et al., 2020).

### Related Diseases and Mechanism of Iron Overload

**FIGURE 2:**
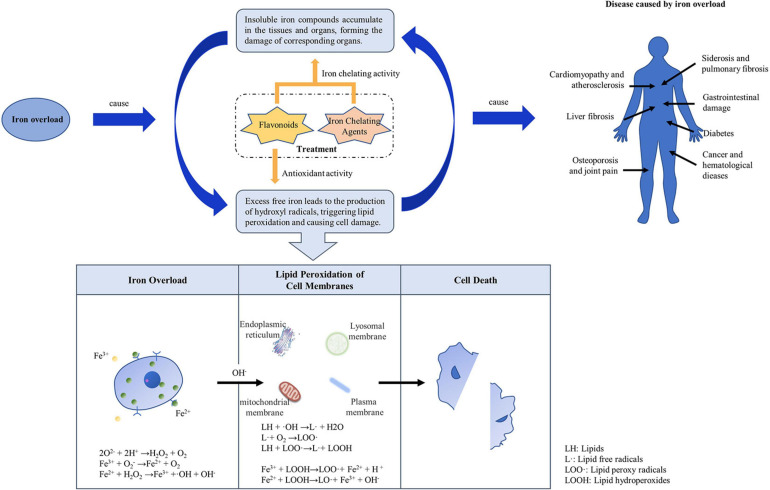
Mechanism of iron overload causing disease.

When there is too much iron in the body, the body will mount a defensive response, but after excess iron exceeds the binding capacity of serum FPN, it becomes labile plasma iron (LPI), labile cellular iron (LCI), or non-transferrin bound iron (NTBI). Unbound free iron leads to the formation of dangerous free radicals, which lead to lipid peroxidation. The accumulation of unstable iron (LPI and LCI) and NTBI will inevitably lead to excessive iron deposition in tissues and organs, ultimately causing multiple target organ damage, seriously affecting the quality of life and survival time of patients. In addition, iron overload affects the electron transfer of oxygen (Ali-Rahmani et al., 2014). Tf continues to transport iron ions to cells, and this may eventually lead to aging, chronic anemia, or cancer (Figure 2).

Excessive iron is widely deposited in the parenchymal cells of some organs and tissues of the human body, resulting in multiple organ function damage. For example, iron ions cannot only act on the gastrointestinal mucosa, causing gastrointestinal damage, but also severely damage hepatocytes, causing lipid peroxidation, cell swelling, and tissue necrosis, leading to liver fibrosis or liver cancer (Jang et al., 2014). On the other hand, inhalation of oxide-containing iron can cause toxic reactions in the respiratory tract. Inhaled iron oxide particles gather in the lungs and are swallowed by macrophages, leading to siderosis (Kim and Wessling-Resnick, 2012). Excessive deposition in the pancreas can cause diabetes, in the heart it can cause cardiomyopathy and atherosclerosis, and in bones and joints it can cause osteoporosis and joint pain. These varied diseases may be due to the ability of different cells to synthesize antioxidants or ferritin. Phytochelators such as flavonoids bind to excess iron, decrease the concentration of iron ions in serum and, due to their antioxidant effects, might reduce iron deposits.

The most direct effect of iron overload on cell damage is caused by lipid peroxidation can triggered by free iron ions. In the presence of free iron ions, hydrogen peroxide (H_2_O_2_) and superoxide (O^2–^) produce OH (hydroxyl radicals) via the Fenton and Haber-Weiss reactions (Choi et al., 2020). OH produced *in vivo* is the most important chemical substance that initiates lipid peroxidation and causes DNA damage (Afsar et al., 2016). It can extract hydrogen from lipids to form lipid free radicals. Lipid free radicals react rapidly with oxygen at a high rate to form lipid peroxyl radicals. Lipid peroxides can continue to extract hydrogen to form other lipid free radicals, which become lipid peroxides themselves, and a series of chain reactions ensues to form a large number of lipid peroxides (Chen et al., 2009). In the presence of Fe^2+^/Fe^3+^, iron ions near membrane lipids react with lipid peroxides to form new lipid peroxides and lipid oxygen free radicals, which further accelerate the chain reaction and eventually lead to cross-linking, polymerization, and inactivation of some lipids and functional macromolecular compounds (Choi et al., 2020). This results in damage to DNA, proteins, and lipids in cells, leading to cancer, hypertension, hyperlipidemia, or atherosclerosis. Antioxidants can block lipid chain autoxidation and provide hydrogen to free radicals, especially lipid peroxides, thus forming stable free radicals that do not initiate or induce further lipid oxidation (Ben Salah et al., 2019). The antioxidant properties of phytochemicals, such as flavonoids, may have a similar effect.

### Treatment of Iron Overload

Iron chelating agents are the main treatment options for iron overload diseases. At present, three iron chelating agents, deferrioxamine, deferiprone, and deferasirox, are mainly used in clinical practice (Yang et al., 2018). However, they are currently only used to treat thalassemia iron overload caused by excessive blood transfusions (Kontoghiorghes and Kontoghiorghe, 2020). In addition to the narrow scope of application, they also have some disadvantages, such as frequent use and high prices, obvious side effects, and poor patient compliance.

With the increased understanding of the pathways related to iron homeostasis in the human body, novel iron chelators have been widely studied. For example, hepcidin supplementation can reduce intestinal iron absorption (Swinkels and Drenth, 2008; Katsarou and Pantopoulos, 2018). Tf extracted from human serum, or genetically engineered (Bobik et al., 2019), can be used as a natural iron-chelating agent to treat iron overload in specific parts of the body. However, as far as current technology is concerned, these are difficult to obtain and have low yields and poor activity, which are sufficient for scientific research but not clinical application. The search for an ideal iron chelator has become a popular pursuit.

Flavonoids, which have the capacity to inhibit reactive oxygen species, scavenge free radicals and regulate iron homeostasis, are less expensive and have fewer side effects, and are promising novel iron chelators (Lesjak and Srai, 2019).

## Flavonoids: A Promising Natural Compound for Iron Overload

Flavonoids are widely distributed in fruits, vegetables, tea, wine, seeds, and plant roots (Batiha et al., 2020). Many flavonoids have antitussive, expectorant, antiasthmatic, and antibacterial properties. In addition, flavonoids exert the same effect as phytoestrogens. They also have iron chelating and antioxidative properties. At present, iron overload is an important killer that endangers human health. Some phytochemicals, such as flavonoids, may provide a basis for new therapeutic approaches.

### The Basic Structure of Flavonoids for Iron Overload

Flavonoids are a type of yellow pigment derived from two benzene rings with phenolic hydroxyl groups connected by three carbon atoms, that is, a series of compounds with C6-C3-C6 as the basic carbon structure. According to the different connection modes of the C3, flavonoids can be divided into several sub-types, including flavones, flavanones, flavonols, flavanols, isoflavones, chalcones, aurones, and anthocyanidins (Huynh et al., 2014; Table 1). Compared with other phytochemicals, flavonoids are highly iron chelation ability and reliable antioxidants. It is speculated that flavonoids can regulate iron metabolism and be used to treat iron overload. Two influential structures of flavonoids treating iron overload were presented as follows.

**TABLE 1:** Classification of flavonoids.

**Classification**	**Basic structure**	**Typical examples**
Basic skeleton		—
	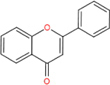	
Flavones		Baicalein, baicalin, apigenin, luteolin
	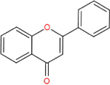	
Flavonols		quercetin, myricetin, kaempferol, rutin
	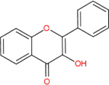	
Flavanones		Naringenin
	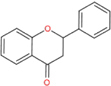	
Flavanols		Catechin
	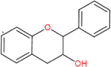	
Isoflavones		Purerarin, genistein
	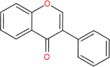	
Chalcones		Corylifolinin
	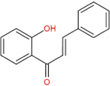	
Aurones		Aureusidin
	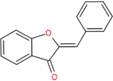	
Anthocyanidins		Cyanidin, delphinidin
	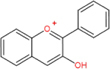	

One is iron chelating properties of flavonoids. Studies have shown that flavonoids contain a variety of iron binding sites (Table 2), such as the 6, 7-dihydroxy structure, B ring catechol, and 2, 3-double bond. However, isolated ketone, hydroxyl, methoxy, or ortho-methoxy groups are not associated with the chelation of iron at all (Mladěnka et al., 2011). The 6, 7-dihydroxy structure is the most effective iron-binding site (Borges et al., 2016). Baicalein and baicalin, which have this structure, have strong iron-chelating properties. However, Vlachodimitropoulou et al. (2011) believe that the most effective is the catechin B ring catechol in quercetin and luteolin, but it may not play an important role in acidic conditions (Mladěnka et al., 2011). In addition, the chelation of metal ions generally requires the presence of 3- or 5-hydroxy groups. Rutin and quercetin contain these structures (Khalili et al., 2015). Furthermore, the 2, 3-double bonds of flavones and flavanols may also be important sites affecting iron binding (Braakhuis, 2019).

**TABLE 2:** Iron binding sites of flavonoids.

	**Basic structure**	**Typical examples**
6,7-dihydroxy		Baicalein, baicalin
	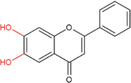	
B ring catechol		Quercetin, luteolin
	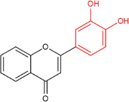	
5-hydroxyl		Rutin, quercetin
	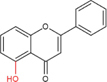	
3-hydroxyl		Rutin, quercetin
	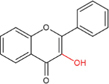	
2,3-double bond		Baicalein, baicalin, rutin, quercetin
	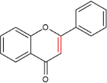	

Another is antioxidant properties of flavonoids. As compounds containing two phenolic hydroxyl groups, flavonoids are effective antioxidants and free radical scavengers (Silva et al., 2020). The more phenolic hydroxyl groups there are, the more sites can be oxidized and the stronger the ability to scavenge oxygen free radicals (Table 2). For example, based on the structure, the oxidation resistance of luteolin is greater than that of apigenin, and that of luteolin is less than quercetin (Supplementary Figure 1).

### The Potential Molecular and Cellular Mechanism of Flavonoids for Iron Overload

**FIGURE 3:**
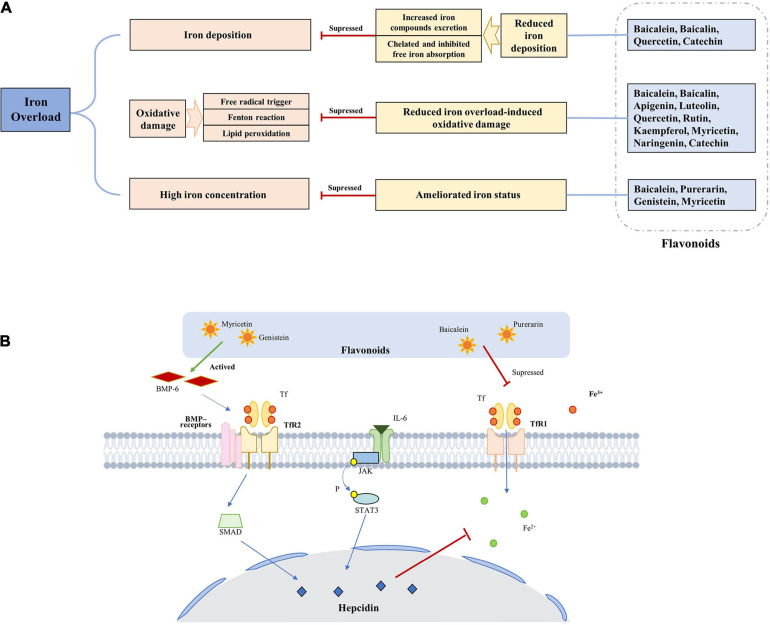
Underlying mechanisms of flavonoids for treating iron overload. **(A)** The basic process of underlying mechanisms. **(B)** The main pathways of flavonoids can ameliorate iron status.

Based on the previous research results, we summarized the underlying mechanisms of flavonoids for treating iron overload according to the following three mechanisms: amelioration of iron status by various proteins and pathways, chelation of iron to reduce iron deposition, and resistance to oxidation to reduce iron overload-induced oxidative damage (Figure 3A).

Flavonoids can ameliorate iron status by various proteins and pathways, which is a indirect way to reduce the saturation of iron (Figure 3B), such as ferritin, Tf, and hepcidin. The mechanism may involve inhibition of the expression of TfR1 and ferroportin 1, promoting the expression of divalent metal transporter 1, increasing hepcidin transcript levels and promoter activity. Furthermore, the regulation of hepcidin and its regulatory elements by flavonoids is related to the STAT3-binding site.

Flavonoids can chelate iron to reduce iron accumulation, which is a direct way to reduce the saturation of iron. On account of flavonoids have iron-binding sites (Table 2) that are the structural basis of iron chelation. According to the relevant literature, baicalein, baicalin, quercetin, and rutin have the strongest iron chelating activity. When the bodily iron is overloaded, flavonoids inhibit the absorption and redistribution of iron in to some extent by chelating iron, thus reducing the iron content. In addition to inhibiting absorption, they can also increase excretion, and may combine with iron to form flavonoid-iron complexes that are excreted through feces.

Flavonoids can resist oxidation to reduce iron overload-induced oxidative damage. Flavonoids are a class of polyphenolic compounds, have strong reducibility, and can be used as antioxidants. The mechanism of action can be summarized in the following three stages: reaction with superoxide anion radical to prevent free radical trigger, inhibition of the Fenton reaction to prevent hydroxyl radical generation, and reaction with lipid peroxidation groups to prevent lipid peroxidation.

## Progress in the Research Regarding Flavonoids for the Treatment of Iron Overload

A number of *in vitro* and *in vivo* experiments have provided compelling evidence that flavonoids have iron chelating activity and strong antioxidant ability, which can reduce the damage caused by iron overload. These experiments indicate that flavonoids may subsequently be used as potential therapies for iron overload syndromes. Herein, we focus on representative flavonoid compounds and summarize the experimental results regarding the treatment of iron overload-related diseases. We also discuss the related mechanisms of plant iron-chelating agents (Table 3).

**TABLE 3:** Progress in the research regarding flavonoids for the treatment of iron overload.

**Classification**	**Compound**	**Model**	**Mechanism**	**Results**	**References**
Flavones	Baicalein	Male Wistar rats	Scavenged radical	Decreased the level of lipid and protein iron overload-induced oxidation	Czapski et al. (2012)
		A mouse model of aplastic anemia with iron overload complication	Up-regulated hepcidin and its regulators (BMP-6, SMAD, and TfR2) at the protein and mRNA levels	Protected iron overload-induced apoptosis and reduced iron deposition	Dijiong et al. (2019)
		A model of UV/Visible spectroscopic studies	Modulation of metal homeostasis and the inhibition of Fenton chemistry	Ameliorated iron status and decreased iron overload-induced oxidation	Perez et al. (2009)
	Baicalin	Male Kunming mice	Be capable of the antioxidant and iron chelation activities	Protected the liver of iron overload	Zhao et al. (2005)
		Hepatocytes CYP2E1	Chelated iron	Decreased iron overload-induced oxidation	Xu et al. (2012)
		A model of Electron Spin Resonance spectra	Facilitated the transfer of electrons from Fe(2+) to dissolved oxygen	Decreased iron overload-induced oxidation	Nishizaki and Iwahashi (2015)
		C6 cells	Positively regulated divalent metal transporter 1 expression and negatively regulated ferroportin 1 expression	Down-regulated iron concentration and decreased iron deposition	Guo et al. (2014)
		Male Wistar rats	Chelated iron and educed the loss of tyrosine hydroxylase-positive cells	Reduced iron deposition in different brain regions and protected dopaminergic neurons	Xiong et al. (2012)
	Apigenin	A375 human melanoma cell line and	Chelated iron, scavenged radical and inhibited lipoxygenase	Decreased iron overload-induced oxidative damage	Danciu et al. (2018)
	Luteolin	A model evaluated the pH effect on the lipid oxidation and polyphenols	Chelated iron and scavenged radical	Decreased iron overload-induced lipid oxidation	Kim and Choe (2018)
Flavonols	Quercetin	MDCK cells	Facilitated chelatable iron shuttling via glucose transport proteins in either direction across the cell membrane	Ameliorated iron status	Vlachodimitropoulou et al. (2011)
		Male Wistar rats	Be capable of the antioxidant and iron chelation activities	Decreased iron overload-induced oxidative damage, hepatotoxicity and nephrotoxicity	Gholampour and Saki (2019)
		Human colon carcinoma cell line HT29 clone 19A	Protected iron overload-induced DNA breaks and oxidized bases	Decreased iron overload-induced oxidative damage	Glei et al. (2002)
		Male specific-pathogen-free C57BL/6J mice	Lowered the iron level particularly in the islet in T2DM mice and abolished partially oxidative stress in pancreatic tissue	Decreased iron overload-induced oxidative damage	Li et al. (2020)
		β-thalassemia major patients	Reduced high sensitivity C-reactive protein, iron, ferritin, and transferrin saturation and increased transferrin	Ameliorated iron status	Sajadi Hezaveh et al. (2019)
		HUVECs	Protected iron overload-induced mitochondrial dysfunction via ROS/ADMA/DDAHII/eNOS/NO pathway	Decreased iron overload-induced cell damage	Chen et al. (2020)
		Male Kunming mice	Inhibited iron overload-induced lipid peroxidation and protein oxidation of liver, decreased hepatic iron and hepatic collagen content, increased the serum non-heme iron level, released iron from liver and finally excrete it through feces	Decreased iron overload-induced oxidative damage, ameliorated iron status, and reduced iron deposition by excreting iron through feces	Kim and Choe (2018)
	Rutin	Male albino rats	Be capable of the antioxidant and iron chelation activities	Decreased iron overload-induced oxidative damage	Aziza et al. (2014)
	Kaempferol	HepG2 cells	Protected arachidonic acid and iron induced ROS	Decreased arachidonic acid and iron overload-induced oxidative damage	Cho et al. (2019)
	Myricetin	SH-SY5Y cells	Reduce iron contents may via inhibiting transferrin receptor 1 (TfR1) expression	Ameliorated iron status	Wang et al. (2017)
		Sprague Dawley male animals rat hepatocytes	Prevented both lipid peroxidation and accumulation of oxidation products in DNA via stimulation of DNA repair processes	Decreased iron overload-induced genotoxicity	Abalea et al. (1999)
Flavanones	Naringenin	Male Wistar rats	Improved antioxidant enzyme activities	Decreased iron overload-induced oxidative damage	Chtourou et al. (2014)
		Male Wistar rats	Scavenged radical	Restores iron overload-induced brain dysfunction	Chtourou et al. (2015)
Flavanols	Catechin	Male ICR mice	Chelated iron and Scavenged reactive oxygen active nitrogen	Decreased arachidonic acid and iron overload-induced oxidative damage	Yang et al. (2019)
		Male Swiss albino mice	Chelated iron and scavenged radical	Decreased iron overload-induced oxidative damage	Chaudhuri et al. (2015)
Isoflavones	Purerarin	Male Kunming mice and ARPE-19 cells	Be associated with regulation of iron-handling proteins, enhancement of the antioxidant capacity, and the inhibition of MAPK and STAT3 activation and the apoptotic pathways under iron overload condition	Decreased iron overload-induced retinal oxidative damage and reduced retinal iron deposition	Song et al. (2020)
		APPswe/PS1ΔE9 transgenic mice	Decreased iron levels and malondialdehyde content, increased glutathione peroxidase and superoxide and reduced inflammatory response markers	Decreased iron overload-induced oxidative damage and inflammatory response markers	Zhang et al. (2018)
	Genistein	HepG2 cells	Be related to the BMP response element or the STAT3-binding site in the Hepcidin promoter	Increased Hepcidin transcript levels and promoter activity	Zhen et al. (2013)

### Flavones

Flavones mostly form glycosides with sugars and are stored in plants. More than 30 types of glycoside belonging to the flavone group have been found, among which baicalein, baicalin apigenin, and luteolin are widely distributed.

#### Baicalein

Baicalein and baicalin are the main compounds isolated from the roots of the traditional Chinese medicinal herb *Scutellaria baicalensis Georgi*, and can be present at the same time. Animal studies have shown that Baicalein is an antioxidant or iron chelator, and it can significantly reduce the oxidation levels of lipids and proteins (Czapski et al., 2012).

One study showed that baicalein could significantly improve white blood cells and hemoglobin in the bone marrow of anemic mice with regenerative disorder, and had a protective effect on apoptosis induced by iron overload. Baicalein had a limited effect on platelet recovery, which was better than deferrioxamine; however, in reducing iron deposition, baicalein is superior to deferrioxamine and its mechanism may be related to the up regulation of hepcidin and its regulators bone morphogenetic protein 6 (BMP-6), SMAD family member 4 (SMAD4), and transferrin receptor 2 (TfR2) at the protein and mRNA levels (Dijiong et al., 2019). Studies by Perez et al. (2009) suggested that baicalin strongly inhibits iron-promoted Fenton chemistry through a combination of chelation and free radical scavenging mechanisms, whereas baicalin only partially protects against free radical damage. Regulation of the metal dynamic balance and inhibition of Fenton chemistry may be two of the mechanisms of herbal medicine.

#### Baicalin

Baicalin is the 7-hydroxyl group of baicalein, followed by glucuronide. After entering the animal body, baicalein is rapidly transformed into baicalin and other metabolites in the blood. Baicalin is not easily absorbed orally and can be absorbed into the blood only after enzymatic hydrolysis into baicalein in the intestine, which is rapidly transformed into baicalin in the body. The structures of baicalin and baicalein are similar and have similar functions.

The results of Zhao et al. (2005) showed that baicalin has a protective effect on the liver of iron-overload mice, and its mechanism may be due to both the antioxidant and chelating iron activity. Xu et al. (2012) found that baicalin inhibits oxidative stress induced by the combination of alcohol and iron via iron chelation.

The antioxidation and iron chelation properties of baicalin may be related to the following reasons: baicalin promotes the transfer of electrons from Fe^2+^ to dissolved oxygen, so that most of the Fe^2+^ is transformed into Fe^3+^, subsequently inhibiting the formation of hydroxyl radicals (Nishizaki and Iwahashi, 2015). In addition, baicalin positively regulates the expression of divalent metal transporter 1 and negatively regulates the expression of ferroportin 1, which reduces the accumulation of iron in different brain regions (Xiong et al., 2012; Guo et al., 2014).

#### Apigenin

The main botanical sources of apigenin stem from *Chamomile*, *Petroselinum crispum*, and *Apium graveolens L.* have the ability to scavenge free radicals, chelate iron, and inhibit lipoxygenase. Compared with other flavonoids (quercetin and kaempferol), it has the characteristics of low toxicity and non-mutagenicity (Simunkova et al., 2019). Danciu et al. (2018) confirmed that apigenin has the ability to scavenge free radicals, chelate iron, and inhibit lipoxygenase. In addition, they found that apigenin from different sources had different apoptosis-promoting and immune-activating potentials.

#### Luteolin

Luteolin is found in various plants in the form of glycosides. These plants have a high content of *Capsicum annuum L.*, *Dendranthema indicum*, *Lonicera japonica Thunb.*, and *Perilla frutescens (L.) Britt*. Luteolin, which has various pharmacological activities, reduces lipid oxidation of emulsions by scavenging radicals and chelating iron (Kim and Choe, 2018).

### Flavonols

Flavonols include more than 60 types of aglycones, among which quercetin and kaempferol are widely distributed, although rutin and myricetin are also common.

#### Quercetin

Quercetin is a flavonoid that is widely distributed in the flowers, leaves, and fruits of plants and is found in large quantities in Chinese medicine, vegetables, fruits, and red wine. It has the ability to scavenge free radicals and chelate iron.

Gholampour et al. (Gholampour and Saki, 2019) found that quercetin could inhibit hepatorenal toxicity induced by ferrous sulfate and reduce the degree of liver and renal tissue injury in rats. Glei et al. (2002) confirmed through experiments that quercetin might reduce the risk of colon cancer by protecting the oxidative damage to DNA induced by iron. Li et al. (2020) approved that quercetin lowered the iron level particularly in the islet in type 2 diabetes (T2DM) mice and abolished partially oxidative stress in pancreatic tissue. In addition, clinical experiments (Sajadi Hezaveh et al., 2019) indicated that quercetin could significantly reduce the saturation of high-sensitivity C-reactive protein, iron, ferritin, and Tf saturation, and increase Tf. Quercetin has also been shown to improve iron status.

The mechanism of quercetin in the treatment of iron overload mainly depends on its antioxidant properties, which reduce lipid peroxidation and iron chelation. In addition, quercetin may reduce iron overload-induced injury through the ROS/ADMA/DDAH and II/eNOS/NO pathways (Chen et al., 2020). It also has the characteristic of iron-shuttling, and a quercetin concentration of <1 μM can facilitate chelatable iron-shuttling through GLUT1 in any direction on the cell membrane (Vlachodimitropoulou et al., 2011). Quercetin and baicalin can release iron from the liver, which is eventually excreted through feces (Zhang et al., 2006).

These factors make it possible for quercetin to be effectively used in chelotherapy under conditions of iron overload. However, an experiment showed that, in contrast to the traditional antioxidant mechanism of quercetin, quercetin has dual effects on the hemoglobin (Hb) redox reaction *in vitro*. Quercetin significantly aggravates Hb–H_2_O_2_-induced protein oxidation at low concentrations and exhibits protective effects at high concentrations, which may provide new insights into the physiological and pharmacological implications of quercetin in iron overload diseases (Lu et al., 2013).

#### Rutin

Quercetin reacts with rutinose to form rutin. It is an effective component of the dried flowers and mature fruits of *Sophora japonica L.* Rutin is a well-known antioxidant and could be an efficient protective agent against iron overload (Aziza et al., 2014).

Aziza et al. (2014) found that the protective effect of rutin on the liver of iron-loaded rats might be related to its antioxidant and metal-chelating activities. One possible mechanism is that the formation of the iron-rutin complex cannot catalyze the conversion of superoxide ions to reactive hydroxyl radicals, which is the main process of the free radical-mediated toxicity of iron overload.

The fruit extract of *Prunus nepalensis Ser.* (Chaudhuri et al., 2015), *Drosera burmanni i* (Ghate et al., 2015), and *Pleurocybella porrigens* (Khalili et al., 2015) contain rutin and other phytochemicals and contribute to their free radical scavenging and iron chelation activity, potentially offering new natural alternatives to treat patients with iron overload. More studies are needed to determine which compounds are responsible for these biological activities.

#### Kaempferol

Kaempferol is mainly derived from the rhizome of *Kaempferia galanga L.* and widely exists in a variety of fruits, vegetables, and beverages. It has attracted attention because of its anti-cancer, anti-inflammatory, anti-oxidative, anti-viral, and other effects.

The relationship between kaempferol and iron absorption remains unclear. Some researchers believe that kaempferol inhibits iron absorption, while Hart and colleagues (Hart et al., 2015, 2020) believe that it promotes iron absorption. These inconsistencies may be related to the concentration of kaempferol and the pH of the environment. Kaempferol, as a plant iron-chelating agent for the treatment of iron overload-related diseases, requires further study.

However, kaempferol has good antioxidant activity. Cho et al. (2019) observed that kaempferol pretreatment could block the production of ROS induced by arachidonic acid and iron, reverse glutathione depletion, and reduce cell death.

#### Myricetin

Myricetin mainly comes from the extract of the leaves, bark, and roots of *Myrica rubra (Lour.)*, and it has been reported to have the biological functions of anti-oxidation, iron absorption inhibition, regulation of hepcidin, and chelation of iron ions.

Studies by Hart et al. (2015) showed that myricetin inhibits iron uptake by Caco-2 cells. Wang et al. (2017) suggested that myricetin reduces iron content by inhibiting the expression of TfR1. A study (Abalea et al., 1999) showed that myricetin antagonizes iron-induced genotoxicity by stimulating the process of DNA repair, preventing lipid peroxidation and the accumulation of oxidation products in DNA, in order to inhibit iron overload produced by liver cancer. In contrast, Mu et al. (2016) demonstrated that myricetin significantly inhibits hepcidin expression *in vitro* and *in vivo* by inhibiting the BMP/SMAD pathway and increasing the expression of FPN and the level of serum iron.

In addition, myricetin is generally shown to be an antioxidant, but sometimes may also be a pro-oxidant. In the presence of ascorbic acid, myricetin has antioxidative ability; in the absence of ascorbic acid, the pro-oxidative activity is dominant, and these two effects are enhanced when it forms a complex with iron (Chobot and Hadacek, 2011).

Although it is unclear whether myricetin can be used as an iron chelator, these results suggest that myricetin may play a key role in regulating iron homeostasis.

### Flavanones

Flavanones are double-bonded hydrogenated derivatives of flavonoids at the C2-3 position, and most of these plant components have hydroxyl or methoxy groups. They exist in free form or in combined forms in the plant kingdom. The most common compound is naringenin.

#### Naringenin

Naringenin mainly originates from the buds of *Prunus yedoensis Mate* and the core and shell of *Anacardium occidentale*. It has antibacterial, anti-inflammatory, anti-cancer, antispasmodic, and cholagogic effects.

Chtourou et al. (2014) suggested that naringenin could increase the activities of antioxidant enzymes and reduce the oxidative damage observed in the cerebral cortex of iron-treated rats. In addition, they also found that it could improve the anxiogenic-like behavior impairment induced by excessive iron in rats, and emphasized that adding this flavonoid to the diet may prevent brain damage associated with iron load (Chtourou et al., 2015). These results suggest that naringin has antioxidant and iron-chelating properties and can protect nerve tissues.

### Flavanols

Flavanols are reduced from flavanonols and can be regarded as flavanonols after the removal of four carboxyl oxygen atoms. Catechin is a common flavanol.

#### Catechin

The derivatives of Flavan-3-ol is catechin, which is a type of active phenolic substance mainly extracted from tea and other natural plants. The catechin in *Prunus nepalensis Ser. (Steud)* (Chaudhuri et al., 2015) and *Phyllostachys nigra Bamboo stems (henosis variety)* (Yang et al., 2019) have been studied, and it was suggested that iron-mediated oxidative stress could be improved, thereby increasing cell survival.

In addition, *Farsetia hamiltonii Royle* (Basu et al., 2019) and *Drosera burmannii Vahl.* (Ghate et al., 2015) catechin have good antioxidant activity, can remove a variety of reactive oxygen and nitrogen, and chelate iron. However, additional research is needed to determine whether catechins could be used to treat iron overload diseases.

### Isoflavones

Isoflavones are a type of flavonoids mainly found in legumes. Daidzein is an isoflavone that has excellent antioxidant properties, although it has been proven unsuitable as a plant iron-chelating agent, as it does not chelate Fe^3+^ (Dowling et al., 2010). Puerarin and genistein may be potential iron chelating agents.

#### Puerarin

Puerarin is a crown-expanding isoflavone derivative separated from the roots of *Pueraria lobata (Willd) Ohwi* and *Pueraria thunbergiana Benth.*

Experimental data generated by Song et al. (2020) confirmed that puerarin reduces the retinal damage caused by iron overload. The possible mechanism might be associated with the regulation of iron-treated proteins, enhancement of antioxidant capacity (Zhang et al., 2018), inhibition of the activation and apoptosis of MAPK, and STAT3 under iron overload (Song et al., 2020).

#### Genistein

Genistein is derived from *Genista tinctoria Linn.* and *Sophora subprostrata Chun et T. Chen* root, which have antioxidant properties.

Zhen et al. (2013) confirmed that genistein treatment of HepG2 cells could increase both hepcidin promoter activity and transcript levels. This may be related to the BMP response element or the STAT3-binding site in the hepcidin promoter.

### Other Flavonoids

There have been few studies on chalcones and aurones associated with iron overload and their basic mechanisms are still poorly understood.

## Discussion

Many flavonoids have specific metal binding sites, and the multiple phenolic hydroxyl structures they contain also have good oxygen reduction properties. Therefore, flavonoids can chelate iron to form iron complexes for excretion and reduce iron accumulation in tissues and organs. At the same time, they strongly inhibit active oxygen and scavenge free radicals, and can reduce lipid peroxidation caused by iron overload, which results in cell damage. Flavonoids have the effect of regulating iron homeostasis and can be used as plant iron chelating agents. Compared with existing iron-chelating agents, they are very safe and inexpensive. In recent s, a large number of studies have shown that flavonoids can play an important role in the regulation of iron overload and are expected to be developed as natural drugs for the treatment of iron homeostasis disorders. Among them, baicalein, baicalin, quercetin, and rutin have shown outstanding results.

Researchers have made great efforts to explore the molecular mechanisms of flavonoids in iron overload. Although some progress has been made in the study of flavonoids in iron overload, research has mainly focused on *in vitro* studies and l sufficient *in vivo* and clinical evidence is lacking, which are important considerations in evidence-based medicine. Furthermore, the use of flavonoids as iron-chelating agents requires risk and benefit assessment and comparison with existing treatments. In this respect, it is difficult to determine whether flavonoids have significant advantages.

It is also worth exploring whether flavonoids that chelate iron and inhibit lipid peroxidation can play an important role in ferroptosis. In addition, flavonoids also face a series of problems, such as low oral utilization rates and poor water solubility. New drug delivery approaches or dissolution methods for flavonoids have become a hot research topic. For example, the use of nanomaterials may be a viable solution strategy (Xia et al., 2011; Kang et al., 2019). The ability of flavonoids to chelate iron is affected by their concentration and environmental pH, and impacts the dosage and conditions of use. These factors should be the focus of subsequent studies (Abotaleb et al., 2018).

Iron overload can affect the clinical course of a variety of diseases and we suggest that patients with chronic and common diseases, such as cancer and hematological diseases, consume fruits, vegetables, and beverages that contain abundant flavonoids to prevent iron overload damage. Although the amount of flavonoids consumed in the diet for achieving a therapeutic effect remains to be determined, this field continues to hold much promise.

## Author Contributions

XW and YL: conceptualization, data curation, and writing – original draft preparation. LH: data curation and investigation. JL and CL: supervision, validation, and writing – review and editing. CS: conceptualization and writing – review and editing. All authors contributed to the article and approved the submitted version.

## Conflict of Interest

The authors declare that the research was conducted in the absence of any commercial or financial relationships that could be construed as a potential conflict of interest.
