# Supplementary figures and images for: Role of Flavonoids in the Treatment of Iron Overload
Source: Front Cell Dev Biol. 2021 Jul 5;9:685364. doi: 10.3389/fcell.2021.685364 (PMC8287860; doi:10.3389/fcell.2021.685364)

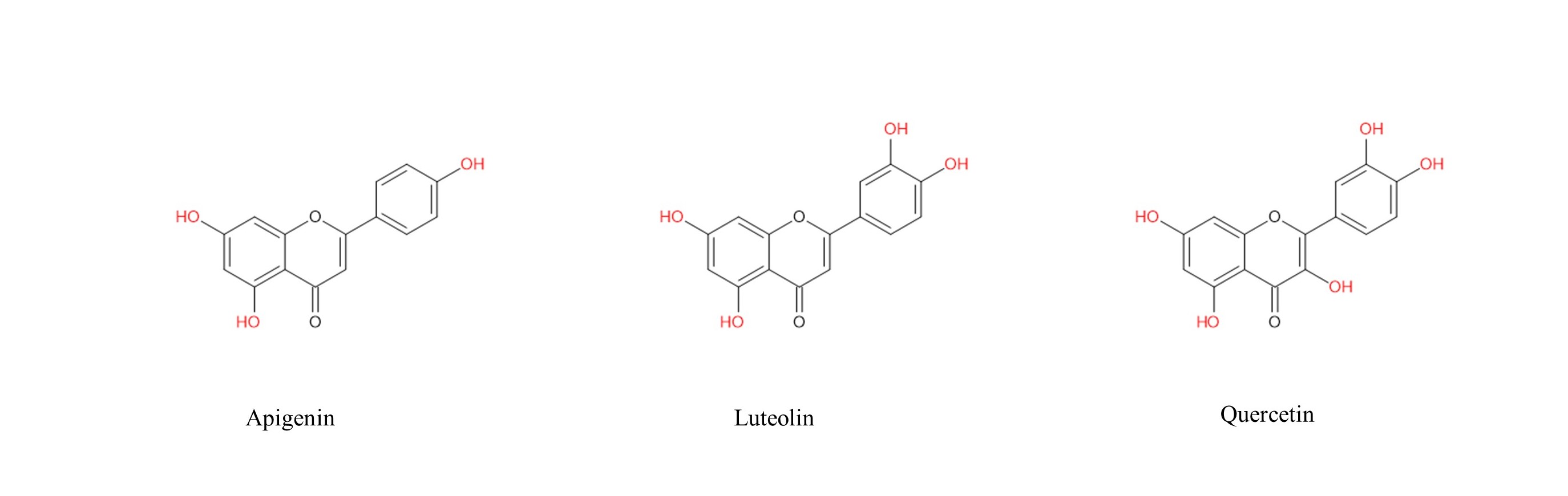

Supplement: Supplementary file 1 [file Image_1.JPEG]
